# A new formula consisting of the five-factor score and earliest vasculitis damage index at diagnosis for predicting poor outcomes of antineutrophil cytoplasmic antibody-associated vasculitis

**DOI:** 10.3389/fmed.2025.1582892

**Published:** 2025-08-06

**Authors:** Jang Woo Ha, Oh Chan Kwon, Yong-Beom Park, Sang-Won Lee

**Affiliations:** ^1^Division of Rheumatology, Department of Internal Medicine, Yongin Severance Hospital, Yonsei University College of Medicine, Yongin, Republic of Korea; ^2^Division of Rheumatology, Department of Internal Medicine, Gangnam Severance Hospital, Yonsei University College of Medicine, Seoul, Republic of Korea; ^3^Division of Rheumatology, Department of Internal Medicine, Yonsei University College of Medicine, Seoul, Republic of Korea; ^4^Institute for Immunology and Immunological Diseases, Yonsei University College of Medicine, Seoul, Republic of Korea

**Keywords:** antineutrophil cytoplasmic antibody-associated vasculitis, five-factor score, vasculitis damage index, prediction, outcomes

## Abstract

**Background:**

This study aimed to investigate whether a new formula consisting of more than two antineutrophil cytoplasmic antibody-associated vasculitis (AAV)-specific indices at diagnosis could predict poor outcomes during follow-up in patients with AAV.

**Methods:**

This study included 323 patients first diagnosed with AAV. AAV-specific indices included the Birmingham vasculitis activity score (BVAS), the five-factor score (FFS), and the earliest vasculitis damage index (eVDI). Poor outcomes included all-cause mortality, end-stage kidney disease (ESKD), cerebrovascular accident (CVA), and acute coronary syndrome (ACS). The four formulas were created by adding each index: BVAS + FFS + eVDI, BVAS + FFS, BVAS + eVDI, and FFS + eVDI.

**Results:**

The median age was 61.0 years (36.2% men). Among the four formulas, FFS + eVDI at AAV diagnosis exhibited the highest area under the curves (AUCs) for all-cause mortality and ESKD in receiver operating characteristic curve analysis. When the optimal cut-off was determined as 4.5 for all-cause mortality and ESKD simultaneously, patients with FFS + eVDI ≥4.5 at AAV diagnosis exhibited significantly higher risks for both all-cause mortality and ESKD, and lower cumulative patients’ and ESKD-free survival rates than those without. in multivariable Cox analyses with other variables at AAV diagnosis, FFS + eVDI at AAV diagnosis was proven to be an independent predictor for all-cause mortality and ESKD during follow-up in patients with AAV.

**Conclusion:**

This study demonstrated that a new formula consisting of FFS and eVDI at AAV diagnosis could effectively predict both all-cause mortality and ESKD during follow-up in patients with AAV.

## 1 Introduction

Antineutrophil cytoplasmic antibody-associated vasculitis (AAV) is one of the two groups of small vessel vasculitides that often affect capillaries and their adjacent arterioles and venules. Unlike immune-complex vasculitis, AAV is characterised by fibrinoid necrotising vasculitis with no or few immune complexes on tissue biopsy (1, 2). Neutrophils play a key role in the pathogenesis of AAV. In particular, their adhesion to vascular endothelial cells, trans-endothelial migration into the extravascular space, and subsequent release of neutrophil components to the extracellular space have been observed as important steps in the development of vascular inflammation (3). According to the clinical features at diagnosis, AAV is divided into three subtypes, microscopic polyangiitis (MPA), granulomatosis with polyangiitis (GPA), and eosinophilic GPA (EGPA) (1, 2). Because AAV can invade almost all major organs including the brain, heart, lungs, and kidneys, it theoretically has the clinical potential to cause severe and life-threatening complications (1, 2, 4, 5). Therefore, to prevent AAV aggravation and exacerbation and maintain its low activity, it is essential to persistently develop and regularly and carefully monitor indicators reflecting and estimating the degree of inflammation and signs of damage of the currently involved major organs (4, 5). In this context, in real clinical settings, the indices of the three aspects of AAV are currently collected at regular intervals, including the Birmingham vasculitis activity score (BVAS) and the five-factor score (FFS) for activity assessment, the vasculitis damage index (VDI) for damage assessment, and the short-form 36 items (SF-36) for function assessment, in addition to diverse laboratory and radiological examinations (6–9). These indices play important roles in determining the current status of vasculitis and establishing future patient-tailored treatment plans. In addition to the primary clinical utility of these indices in reflecting the current and diverse cross-sectional vasculitis status of AAV, the positive aspects of the predictive ability of BVAS, FFS, and VDI assessed at AAV diagnosis for poor outcomes of AAV have been continuously unveiled and suggested to date (10–12). Furthermore, based on their clinical aspects, it could be inferred that a new formula consisting of more than two AAV-specific indices would have a significantly better predictive ability for poor outcomes of AAV than each single index. However, no study has compared the clinical efficacy between an index combining more than two AAV-specific indices and each single index for predicting poor outcomes in patients with AAV. Hence, in this study, we developed a new formula consisting of more than two AAV-specific indices and investigated whether the new formula could effectively predict poor outcomes in patients with AAV.

## 2 Methods

### 2.1 Patients

This study included 323 patients with AAV enrolled in the Severance Hospital ANCA-associated VasculitidEs (SHAVE) cohort, an observational single-centre cohort of Korean patients with AAV. The inclusion criteria of this study were the same as those of the cohort of AAV patients as follows: (i) patients who had been diagnosed with AAV for the first time by the specialised Rheumatologists in this hospital from November 2005 to March 2024; (ii) patients whose AAV diagnosis was made based on the classification criteria for AAV proposed by the American College of Rheumatology (ACR) in 1990, the classification algorithm suggested by the European Medicine Agency in 2007, and the revised nomenclature of systemic vasculitides established by the Chapel Hill Consensus Conference in 2012 (2, 3, 13, 14); (iii) patients who also met the classification criteria for MPA, GPA, and EGPA proposed by a joint group of the ACR and the European Alliance of Associations for Rheumatology in 2022 (15–17); (iv) patients who had the medical records sufficiently documented for not only collecting clinical, laboratory, radiological, and histological data at AAV diagnosis and during follow-up (15–18); (v) patients in whom the tests for ANCA were performed within 4 weeks before or after AAV diagnosis; (vi) patients who had been followed up for at least 6 months or more after AAV diagnosis; (vii) patients who had no concomitant serious medical conditions mimicking AAV such as malignancies or severe infectious diseases requiring hospitalisation at AAV diagnosis (19, 20); (viii) patients who had not received immunosuppressive drugs for AAV treatment within 4 weeks before AAV diagnosis.

### 2.2 Ethical statement

This study was approved by the Institutional Review Board (IRB) of Severance Hospital (Seoul, Korea, IRB No. 4–2020-1071), and conducted in accordance with the Declaration of Helsinki. Given the retrospective design of the study and the use of anonymised patient data, the requirement for written informed consent was waived.

### 2.3 Clinical data at AAV diagnosis

Pertaining to variables at AAV diagnosis, age, sex, body mass index (BMI), and smoking history were obtained as demographic data. The AAV subtype, ANCA type and positivity, and AAV-specific indices including BVAS and FFS were collected. Laboratory results, particularly, erythrocyte sedimentation rate (ESR), and C-reactive protein (CRP) levels, were recorded. Hypertension, type 2 diabetes mellitus (T2DM), and dyslipidaemia were also reviewed as comorbidities (Table 1).

**Table 1:** Characteristics of patients at AAV diagnosis.

Variables	Values
Demographic data	
Age (years)	61.0 (50.0–69.0)
Male sex [*N*, (%)]	117 (36.2)
BMI (kg/m^2^)	22.5 (20.3–24.8)
Ex-smoker [*N*, (%)]	9 (2.8)
AAV subtype [*N*, (%)]	
MPA	184 (57.0)
GPA	76 (23.5)
EGPA	63 (19.5)
ANCA type and positivity [*N*, (%)]	
MPO-ANCA (or P-ANCA) positivity	226 (70.0)
PR3-ANCA (or C-ANCA) positivity	51 (15.8)
Both ANCA positivity	14 (4.3)
ANCA negativity	60 (18.6)
AAV-specific indices	
BVAS	12.0 (7.0–18.0)
FFS	1.0 (0–2.0)
eVDI^*^	3.0 (2.0–4.0)
New equations using AAV-specific indices	
BVAS + FFS + eVDI	16.0 (10.0–23.0)
BVAS + FFS	13.0 (8.0–19.0)
BVAS + eVDI	14.0 (9.0–21.0)
FFS + eVDI	4.0 (3.0–5.0)
Acute phase reactants	
ESR (mm/h)	59.0 (22.0–96.0)
CRP (mg/L)	11.9 (1.6–61.0)
Laboratory results	
White blood cell count (/mm^3^)	9,240.0 (6,430.0 − 12,920.0)
Haemoglobin (g/dL)	11.5 (9.6–13.3)
Platelet count (× 1,000/mm^3^)	295.0 (227.0–387.0)
Fasting glucose (mg/dL)	101.0 (90.0–121.0)
Blood urea nitrogen (mg/dL)	17.6 (12.6–30.9)
Serum creatinine (mg/dL)	0.9 (0.7–1.7)
Serum total protein (g/dL)	6.8 (6.1–7.3)
Serum albumin (g/dL)	3.7 (3.1–4.3)
Comorbidities [*N*, (%)]	
T2DM	81 (25.1)
Hypertension	132 (40.9)
Dyslipidaemia	53 (16.4)

Values are expressed as a median (25 percentile, 75 percentile) or *N* (%). eVDI*, the earliest VDI was defined as follows: the first VDI assessed at more than 3 months after AAV diagnosis or at more than 3 months after the first presentation of AAV-related manifestations. AAV, ANCA-associated vasculitis; ANCA, antineutrophil cytoplasmic antibody; BMI, body mass index; MPA, microscopic polyangiitis; GPA, granulomatosis with polyangiitis; EGPA, eosinophilic granulomatosis with polyangiitis; MPO, myeloperoxidase; P, perinuclear; PR3, proteinase 3; C, cytoplasmic; BVAS, the Birmingham vasculitis activity score; FFS, the five-factor score; eVDI, the earlies vasculitis damage index; ESR, erythrocyte sedimentation rate; CRP, C-reactive protein; T2DM: type 2 diabetes mellitus.

### 2.4 Earliest vasculitis damage index

The earliest VDI (eVDI), one of the AAV-related indices, was also obtained as follows: a total score of eVDI should be evaluated three or more months after AAV diagnosis; however, another total score of eVDI which is assessed within 3 months after AAV diagnosis will be accepted only if two conditions as described in our previous study are met: one is that more than 3 months have elapsed since the onset of AAV-related symptoms, and the other is that the damage has initiated or worsened from the onset of AAV itself (11) (Supplementary Figure 1). Although there was a time gap between the time of assessment of BVAS and FFS and that of eVDI in this study, we considered that eVDI was measured within 3 months of AAV diagnosis and that a total score of eVDI does not decrease despite improvement (8, 11). Therefore, in this study, like BVAS and FFS at AAV diagnosis, ‘the baseline or initial eVDI’ was considered ‘eVDI at AAV diagnosis’ for convenience.

### 2.5 ANCA tests

An immunoassay was performed for measuring the titres of myeloperoxidase (MPO)-ANCA and proteinase 3 (PR3)-ANCA, whereas, an indirect immunofluorescence assay was used for detecting the presence of perinuclear (P)-ANCA and cytoplasmic (C)-ANCA as well (21). In the present study, the results of both ANCA tests, an immunoassay, and an indirect immunofluorescence assay were recognized (15–18).

### 2.6 Clinical data during follow-up

Regarding variables during follow-up after AAV diagnosis, all-cause mortality, end-stage kidney disease (ESKD), cerebrovascular accident (CVA), and acute coronary syndrome (ACS) during follow-up after AAV diagnosis were evaluated as poor outcomes of AAV in the present study. When ESKD, CVA, or ACS occurred before AAV diagnosis, they were not considered poor outcomes of AAV. All-cause mortality was defined as death due to any cause; however, death resulting from traffic accidents and natural disasters were not included in this study. ESKD was defined as a renal status requiring renal replacement therapy (22). CVA was defined as stroke including cerebrovascular thrombosis and/or haemorrhage (23). ACS included ST-elevation myocardial infarction (STEMI), non-STEMI (NSTEMI), and unstable angina (24). The follow-up duration based on each poor outcome was defined as the period from the time of AAV diagnosis to the time when each poor outcome occurred in patients with each poor outcome, and conversely, to the last visit in patients without. The number of patients with AAV who received glucocorticoids and immunosuppressive drugs during follow-up after AAV diagnosis was also counted.

### 2.7 New formulas consisting of more than two AAV-specific indices

In this study, given the convenience in real clinical practice, we arbitrarily created four new formulas consisting of more than two AAV-specific indices at AAV diagnosis by adding more than two variables and compared their clinical potential as predictors of poor outcomes in patients with AAV. The four groups of formulas were as follows: (i) BVAS + FFS + eVDI, (ii) BVAS + FFS, (iii) BVAS + eVDI, and (iv) FFS + eVDI.

### 2.8 Statistical analysis

All statistical analyses were performed using SPSS version 26 (IBM Corporation, Armonk, NY, USA) for Windows (Microsoft Corporation, Redmond, WA, USA). Continuous and categorical variables were expressed as medians (interquartile ranges [IQR], 25–75 percentiles), and numbers (percentages). Significant differences between two categorical variables were analysed using the Chi-square and Fisher’s exact tests. The Mann–Whitney U test was used to compare significant differences between two continuous variables. The correlation coefficients (r) between two variables were determined using Pearson’s correlation analysis. A significant area under the curve (AUC) was determined using a receiver operating characteristic (ROC) curve analysis. The cut-off was extrapolated by performing ROC curve analysis and selected as the value with the maximum sum of sensitivity and specificity. A multivariable Cox proportional hazard model using variables with *p* < 0.1 in a univariable Cox analysis was performed to obtain a hazard ratio (HR) during follow-up. A comparison of the cumulative survival rates between the two groups was performed using Kaplan Meier survival analysis with the log-rank test. *p* < 0.05 was considered to be statistically significant.

## 3 Results

### 3.1 Characteristics of patients at AAV diagnosis

The median age of the 323 patients, 36.2% of whom were men, was 61.0 years, and nine had ever smoked but were not current smokers. Among them, 184 (57.0%), 76 (23.5%), and 63 (19.5%) patients were diagnosed with MPA, GPA, and EGPA, respectively. MPO-ANCA (or P-ANCA), and PR3-ANCA (or C-ANCA) were positive in 226 (70.0%), and 51 (15.8%) patients, respectively. The median BVAS, FFS, and eVDI were 1 2.0, 1.0, and 3.0, respectively. Additionally, the median values of BVAS + FFS + eVDI, BVAS + FFS, BVAS + eVDI, and FFS + eVDI formulas were 16.0, 13.0, 14.0, and 4.0, respectively. The median ESR and CRP levels were 50.0 mm/h, and 11.9 mg/L, respectively. Among the patients, 81 (25.1%), 132 (40.9%), and 53 (16.4%) were diagnosed with T2DM, hypertension, and dyslipidaemia before or at the time of AAV diagnosis: those who were diagnosed with these comorbidities after AAV diagnosis were excluded (Table 1).

### 3.2 Characteristics during follow-up

Among the 323 patients, 45 (13.9%) died for a median follow-up duration of 53.7 months based on all-cause mortality. Additionally, 52 (16.1%), 21 (6.5%), and 13 (4.0%) patients progressed to ESKD, CVA, and ACS, respectively, during the follow-up duration based on each poor outcome after AAV diagnosis. Glucocorticoids were administered to 305 (94.4%) patients, and the most commonly administered immunosuppressive drug was cyclophosphamide (51.7%), followed by azathioprine (50.8%). Whereas, 59 (18.3%), and 79 (24.5%) patients received rituximab and mycophenolate mofetil, respectively (Table 2).

**Table 2:** Characteristics of patients with AAV during follow-up.

Variables	Values
Poor outcomes [*N*, (%)]	
All-cause mortality	45 (13.9)
ESKD	52 (16.1)
CVA	21 (6.5)
ACS	13 (4.0)
Follow-up duration based on each poor outcome (months)	
All-cause mortality	53.7 (18.6–89.4)
ESKD	43.3 (10.7–81.0)
CVA	48.2 (12.9–86.9)
ACS	50.4 (17.0–86.6)
Medications [*N*, (%)]	
Glucocorticoids	305 (94.4)
Cyclophosphamide	167 (51.7)
Rituximab	59 (18.3)
Mycophenolate mofetil	79 (24.5)
Azathioprine	164 (50.8)
Tacrolimus	24 (7.4)
Methotrexate	39 (12.1)

Values are expressed as a median (25–75 percentile) or *N* (%). ESKD, end-stage kidney disease; CVA, cerebrovascular accident; ACS, acute coronary syndrome.

### 3.3 Area under the curve of four formulas for each poor outcome

**Figure 1:**
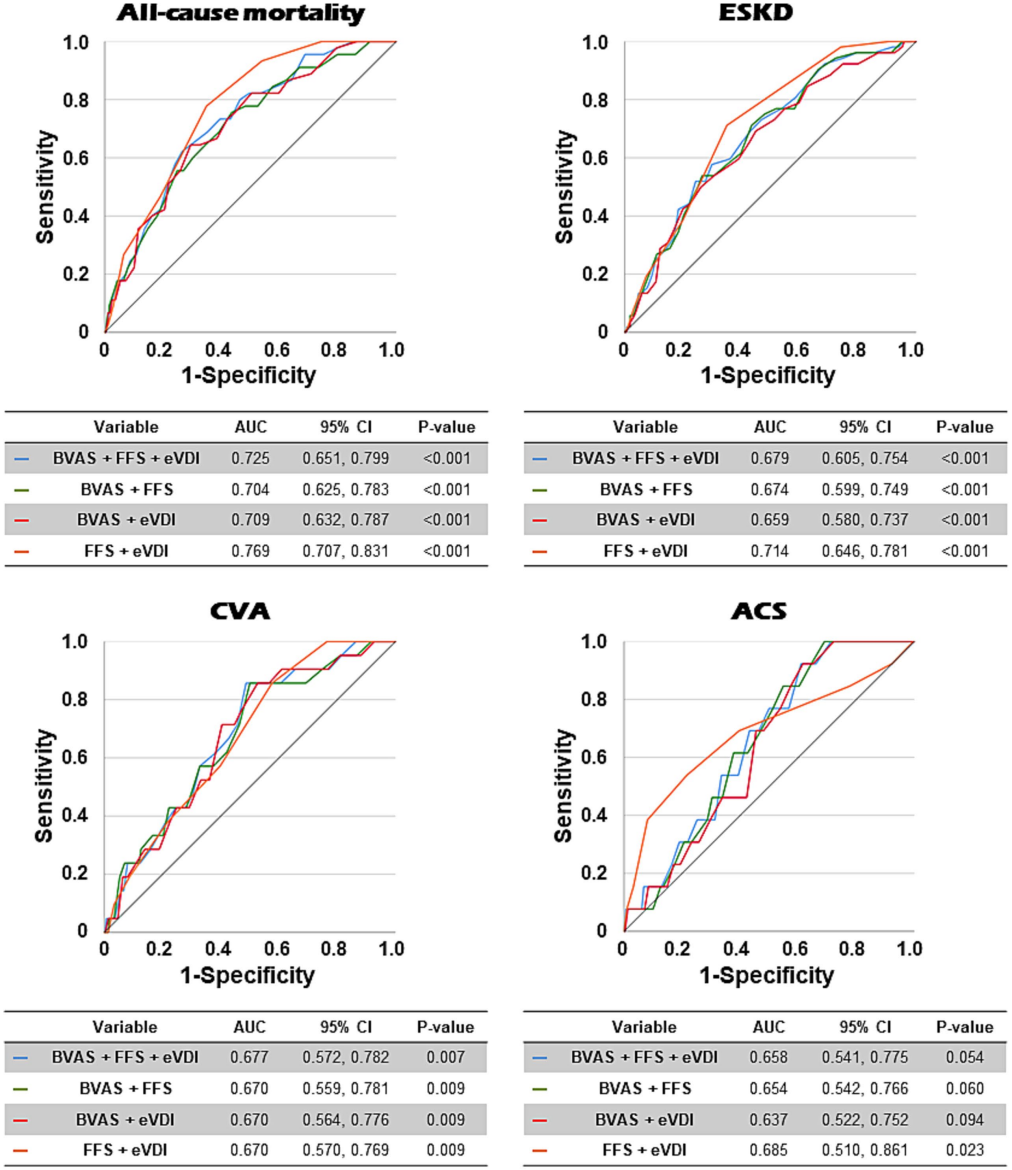
Comparison of AUCs of four formulas for all-cause mortality, ESKD, CVA, and ACS in patients with AAV in ROC curve analysis. The formula of FFS + eVDI exhibited the highest AUCs for all-cause mortality and ESKD, but not for CVA. AUC: area under the curve; ESKD: end-stage kidney disease; CVA: cerebrovascular accident; ACS: acute coronary syndrome; AAV: ANCA-associated vasculitis; ANCA: antineutrophil cytoplasmic antibody; ROC: receiver operating characteristic; CI: confidence interval; BVAS: the Birmingham vasculitis activity score; FFS: the five-factor score; eVDI: the earliest vasculitis damage index.

In ROC curve analyses, among the AUCs of the four formulas for all-cause mortality, the formula of FFS + eVDI exhibited the highest AUC [area 0.769, 95% confidence interval (CI) 0.707, 0.831], followed by the formula of BVAS + FFS + eVDI (area 0.725, 95% CI 0.651, 0.799). Among the AUCS of the four formulas for ESKD, the formula of FFS + eVDI also showed a significantly higher AUC (area 0.714, 95% CI 0.646, 0.781) compared to the remaining three formulas. Regarding the AUCs of the four formulas for CVA and ACS, however, the results of ROC curve analyses for both CVA and ACS showed AUCs of less than 0.7, making it difficult to expect high clinical utility (Figure 1). Based on the results of these analyses, among the four formulas, the formula of FFS + eVDI was selected and its predictive potential for all-cause mortality and ESKD among the four poor outcomes of AAV was analysed in this study.

### 3.4 Cut-offs and RRs of the formula of FFS + eVDI for all-cause mortality and ESKD

**Figure 2:**
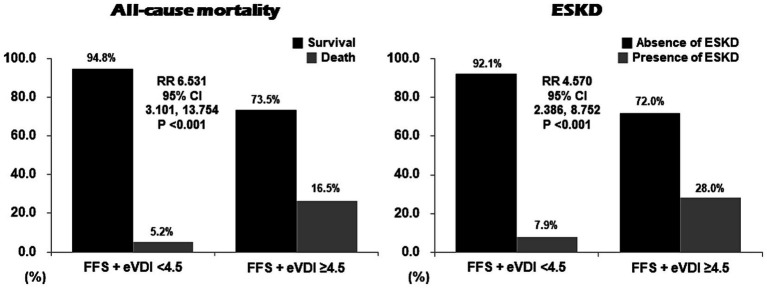
Evaluation of optimal cut-offs and RRs of the formula of FFS + eVDI for all-cause mortality and ESKD in patients with AAV. When the cut-off was extrapolated as 4.5 for all-cause mortality as well as ESKD, patients with FFS + eVDI ≥4.5 exhibited significantly higher risks for both poor outcomes during follow-up. RR: relative risk; FFS: the five-factor score; eVDI: the earliest vasculitis damage index; ESKD: end-stage kidney disease; AAV: ANCA-associated vasculitis; ANCA: antineutrophil cytoplasmic antibody; CI: confidence interval.

Using ROC curve analyses, the optimal cut-off of the formula of FFS + eVDI for all-cause mortality (sensitivity was 77.8%, and specificity was 65.1%) was determined as 4.5. The cut-off of the formula of FFS + eVDI for ESKD (sensitivity was 71.2%, and specificity was 64.9%) was also determined as 4.5. When dividing patients into two groups according to FFS + eVDI ≥4.5, we found that patients with FFS + eVDI ≥4.5 at AAV diagnosis exhibited significantly higher risks for both all-cause mortality (16.5% vs. 5.2%; RR 6.531, 95% CI 3.101, 13.754, *p* < 0.001) and ESKD (28.0% vs. 7.9%; RR 4.570, 95% CI 2.386, 8.752, p < 0.001) compared to those without (Figure 2).

### 3.5 Cumulative survival rates according to FFS + eVDI ≥4.5

**Figure 3:**
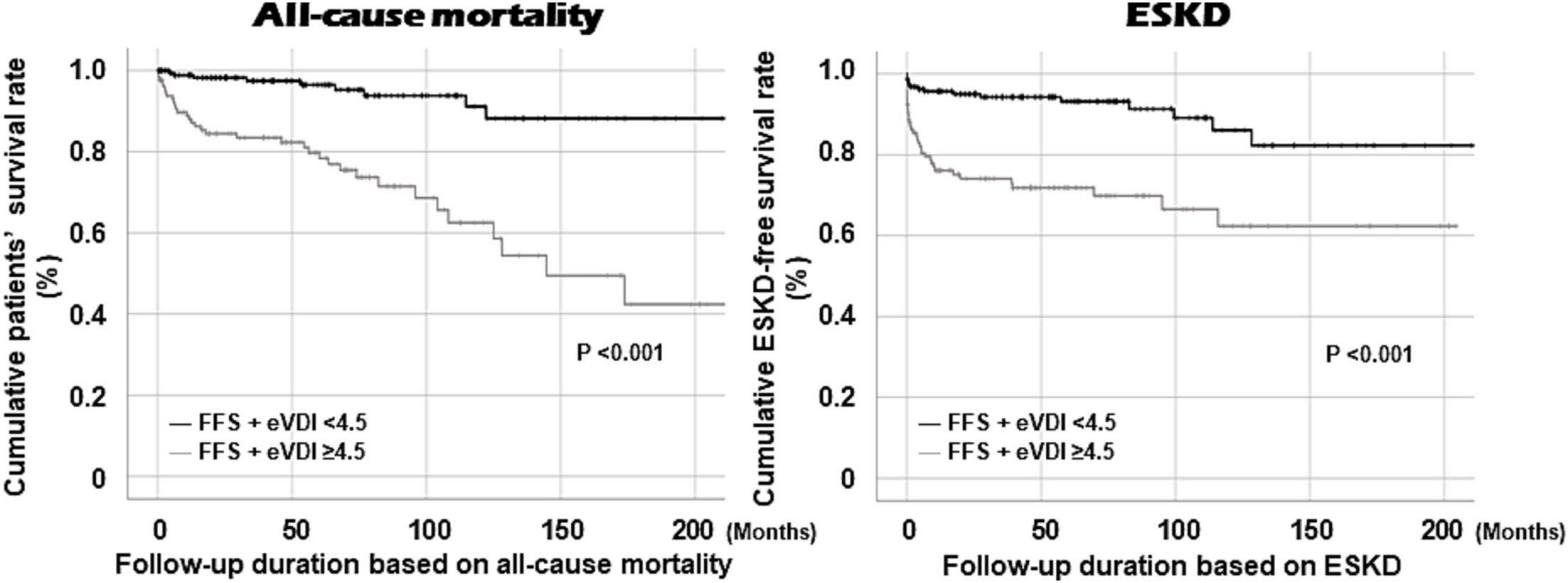
Comparison of cumulative survival rates. Patients with FFS + eVDI ≥4.5 exhibited significantly lower cumulative patients’ and ESKD-free survival rates than those with FFS + eVDI <4.5. ESKD, end-stage kidney disease; FFS, the five-factor score; eVDI, the earliest vasculitis damage index; AAV, ANCA-associated vasculitis; ANCA, antineutrophil cytoplasmic antibody.

Patients with FFS + eVDI ≥4.5 at AAV diagnosis exhibited a significantly lower cumulative patients’ survival rate than those without. Similarly, patients with FFS + eVDI ≥4.5 at AAV diagnosis showed a significantly reduced cumulative ESKD-free survival rate compared to patients without (Figure 3).

### 3.6 Cox proportional analyses for all-cause mortality and ESKD

First, in univariable Cox proportional hazard analysis for all-cause mortality, age (HR 1.060), male sex (HR 1.988), ESR (HR 1.008), CRP (HR 1.008), haemoglobin (HR 0.752), blood urea nitrogen (HR 1.013), serum creatinine (HR 1.147), serum total protein (HR 0.577), serum albumin (HR 0.385), dyslipidaemia (HR 2.039), and FFS + eVDI (HR 1.538) at AAV diagnosis were significantly associated with all-cause mortality during follow-up (Supplementary Table 1). In multivariable Cox analysis including only the variables with significant HRs in univariable analysis, serum albumin (HR 0.514, 95% CI 0.285, 0.928) and FFS + eVDI (HR 1.355, 95% CI 1.128, 1.628) at AAV diagnosis were significantly and independently associated with all-cause mortality during follow-up in patients with AAV. Both age and male sex also tended to be associated with all-cause mortality; however, no statistical significance was observed (Table 3). Next, in univariable Cox proportional hazard analysis for ESKD, age (HR 1.025), BMI (HR 0.883), MPO-ANCA (or P-ANCA) positive (HR 2.747), haemoglobin (HR 0.664), blood urea nitrogen (HR 1.031), serum creatinine (HR 1.533), serum albumin (HR 0.580), hypertension (HR 2.380), and FFS + eVDI (HR 1.406) at AAV diagnosis exhibited the significant association with ESKD during follow-up (Supplementary Table 2). In multivariable Cox analysis including only the variables with significant HRs in univariable analysis, BMI (HR 0.889, 95% CI 0.801, 0.987), serum creatinine (HR 1.451 95% CI 1.275, 1.651) and FFS + eVDI (HR 1.289, 95% CI 1.070, 1.553) at AAV diagnosis were significantly and independently associated with ESKD during follow-up in patients with AAV (Table 3). Meanwhile, the results of comparative analyses of variables at diagnosis according to all-cause mortality or ESKD during follow-up also supported the results of univariable Cox analysis for each poor outcome in AAV patients (Supplementary Tables 3, 4).

**Table 3:** Multivariable Cox proportional hazard analyses of variables at diagnosis with statistical significance in univariable Cox analysis for each poor outcome during follow-up in AAV patients.

Variables	HR	95% CI	*p* value
All-cause mortality			
Age	1.027	0.998, 1.057	0.073
Male sex	1.875	0.958, 3.671	0.067
ESR	0.994	0.984, 1.005	0.268
CRP	1.001	0.994, 1.008	0.718
Haemoglobin	0.923	0.749, 1.138	0.454
Blood urea nitrogen	1.005	0.992, 1.019	0.442
Serum creatinine	1.001	0.817, 1.227	0.989
Serum total protein	0.973	0.885, 1.071	0.578
Serum albumin	0.514	0.285, 0.928	0.027
Dyslipidaemia	1.647	0.803, 3.379	0.173
FFS + eVDI	1.355	1.128, 1.628	0.001
ESKD			
Age	1.018	0.992, 1.044	0.177
BMI	0.889	0.801, 0.987	0.028
MPO-ANCA (or P-ANCA)	1.540	0.710, 3.338	0.274
Haemoglobin	0.929	0.775, 1.113	0.424
Blood urea nitrogen	1.006	0.993, 1.018	0.374
Serum creatinine	1.451	1.275, 1.651	<0.001
Serum albumin	0.930	0.560, 1.544	0.778
Hypertension	1.551	0.833, 2.888	0.166
FFS + eVDI	1.289	1.070, 1.553	0.008

AAV, ANCA-associated vasculitis; ANCA, antineutrophil cytoplasmic antibody; HR, hazard ratio; CI, confidence interval; ESR, erythrocyte sedimentation rate; CRP, C-reactive protein; FFS, the five-factor score; eVDI, the earlies vasculitis damage index; BMI, body mass index; MPO, myeloperoxidase; P, perinuclear.

## 4 Discussion

In this study, we developed a new formula consisting of FFS and eVDI by adding the two variables and demonstrated its clinical usefulness in predicting poor outcomes in immunosuppressive-naïve patients who were newly diagnosed with AAV. The results of this study are summarised as follows. First, we evaluated the clinical significance of four new formulas consisting of more than two AAV-specific indices. Second, using ROC curve analyses, we compared their predictive potential for the four poor outcomes of AAV and found that the formula of FFS + eVDI at AAV diagnosis showed the highest and statistically significant AUCs for all-cause mortality and ESKD during follow-up in patients with AAV. Whereas, those for CVA and ACS were less useful. Third, the optimal cut-off of the formula of FFS + eVDI was determined to be the same at 4.5 for both all-cause mortality and ESKD. Fourth, patients with FFS + eVDI ≥4.5 at AAV diagnosis exhibited significantly higher risks for both all-cause mortality and ESKD than those without. Fifth, patients with FFS + eVDI ≥4.5 at AAV diagnosis exhibited significantly lower cumulative patients’ and ESKD-free survival rates than those without. Last, in multivariable Cox analyses, FFS + eVDI at AAV diagnosis was significantly associated with not only all-cause mortality but also ESKD during follow-up in patients with AAV. In this study, we demonstrated for the first time the clinical utility of the formula consisting of both FFS and eVDI at AAV diagnosis in predicting the major poor outcomes of AAV such as all-cause mortality, and ESKD.

It was thought that the clinical value of the formula consisting of FFS + eVDI could be further increased if it could also predict CVA and ACS, which are important and fatal complications of AAV (25, 26). However, FFS + eVDI at AAV diagnosis for CVA and ACS during follow-up was not included in further analyses because it showed relatively low AUCs compared with those for all-cause mortality or ESKD, despite statistical significance in ROC curve analysis. Instead, we used ROC curve analyses and obtained the cut-offs of FFS + eVDI at AAV diagnosis for CVA and ACS at 3.5 (sensitivity was 85.7%, and specificity was 42.4%), and 5.5 (sensitivity was 53.8%, and specificity was 78.4%), respectively. In Kaplan Meier survival analysis, patients with FFS + eVDI ≥3.5 at AAV diagnosis exhibited a significantly lower cumulative CVA-free survival rate than those with FFS + eVDI <3.5 (*p* = 0.034). Similarly, patients with FFS + eVDI ≥5.5 at AAV diagnosis showed a significantly reduced cumulative ACS-free survival rate compared to patients with FFS + eVDI <5.5 (*p* = 0.003) (Supplementary Figure 2). However, although statistically significant, the survival rates between the two groups for CVA and ACS did not differ significantly enough to be clinically applicable. In this study, the number of patients who experienced CVA or ACS was not large enough, so the instability of significance according to the analysis was observed. Therefore, for the AAV cohort with a high frequency of CVA or ACS, we believe that it is worth considering deriving a new formula consisting of more than two AAV-specific indices at AAV diagnosis for anticipating the occurrence of CVA or ACS during follow-up.

In terms of the predictive potential for all-cause mortality, in ROC curve analysis, BVAS, FFS, and eVDI showed significant AUCs in ROC curve analysis comparable to FFS + eVDI; however, when comparing the four variables, FFS + eVDI exhibited the highest AUC. Conversely, in terms of the predictive potential for ESKD, in ROC curve analysis, FFS would rather show a slightly higher AUC than FFS + eVDI (Supplementary Figure 3). That is, the advantage of the formula of FFS + eVDI does not seem to be evident compared to other individual AAV-specific indices. Nevertheless, the reason for developing a new formula that includes more than two variables is that it provides stability that can buffer the rapid changes of a single variable (12). Additionally, the differences between the subitems that make up each variable play a complementary role. For example, in terms of eVDI, variables that are not included in FFS but are included in eVDI and affect death or ESKD, such as hypertension, T2DM, lung fibrosis, and osteoporosis, are included (8). On the other hand, in terms of FFS, variables that are more AAV-specific and friendly, such as gastrointestinal and heart involvement, are included (7). Therefore, it is thought that it would be clinically advantageous to use a formula that includes two or more associated variables rather than a single variable unless there is a clear difference in the clinical utility for predicting poor outcomes among individual variables.

Theoretically, BVAS + FFS + eVDI was expected to have a greater ability to predict death or ESKD than FFS + eVDI, but the results revealed it was not. We wondered what was the reason for a decrease in the ability to predict poor outcomes of AAV when BVAS was added to the formula of FFS + eVDI, and inferred that it would be owing to the differences in persistency and reversibility of the subitems among the indices. FFS and eVDI mainly contain relatively chronic and non-reversible sub-items, whereas, BVAS contains a considerable number of acute and reversible sub-items (6–8). In particular, among the subitems of BVAS, higher scores are assigned to ‘new’ or ‘worsening’ ones than to persistent ones (6). Therefore, it could be reasonably speculated that the acute and reversible subitems of BVAS may have the potential to be improved, which may paradoxically impose a negative effect on the ability of the formula of BVAS + FFS + eVDI to predict future poor outcomes of AAV, compared to FFS + eVDI.

The risk factors of all-cause mortality and ESKD in AAV patients are being understood and applied to real clinical practice by classifying them into three categories: general, AAV-specific, and inflammatory risk factors (27–29). Because this study was conducted to derive a poor prognosis predictive formula including two or more of the three AAV-specific indices, general and inflammatory risk factors could not be included in the formula of FFS + eVDI. However, we expect that these issues might be offset in two aspects. First, in multivariable COX proportional hazard analysis, FFS + eVDI showed statistically significant predictive ability even after the adjustment of age, male sex, and comorbidities such as T2DM, hypertension, and dyslipidaemia. Second, except for male sex for all-cause mortality, the items of FFS and eVDI are comprehensively complementary: FFS includes the item of an age of 65 years or older, and eVDI includes the items of diastolic hypertension and T2DM (7, 8).

Given the differences in the pathophysiology and the patterns of the corresponding clinical manifestations and poor outcomes between MPA/GPA and EGPA, the need for the exclusion of EGPA patients from this study could have been raised. To provide the answer to this issue, we conducted an additional analysis to compare the AUCs of FFS + eVDI according to all-cause mortality and ESKD between patients with MPA/GPA/EGPA and those with MPA/GPA. Although the incidence rates of mortality and progression to ESKD slightly differed; however, no significant differences between the two groups were observed (Supplementary Figure 4). In real clinical settings, we occasionally encounter patients meeting the criteria for both MPA and EGPA or GPA and EGPA. Moreover, we also encounter patients of whom AAV subtypes classified at diagnosis alter to other AAV subtypes during follow-up. Therefore, we believe that we should also consider the merits of predictive factor discovery studies that include patients with all three AAV subtypes, as long as there is no error due to obvious differences in the results.

The strength of this study is that it derived and demonstrated a formula for predicting all-cause mortality and progression to ESKD using only AAV-specific indicators, excluding various existing risk factors, and further, its independence was also proven in multivariable Cox analysis with traditional and inflammatory risk factors for all-cause mortality as well as ESKD progression.

### 4.1 Limitations

This study has several limitations. The most critical limitation is the time points of assessment of FFS and that of eVDI were different. Therefore, it was not easy to directly analyse the relationship between other laboratory results at AAV diagnosis and eVDI. Moreover, it seemed impossible to control the administration of immunosuppressive drugs after AAV diagnosis despite close to the time of diagnosis. However, given the principle that a total score of eVDI does not decrease even when improvement occurs, this minor time gap was unlikely to have a significant clinical impact on the results of this study (8, 11). Another limitation is that, although cardiovascular outcomes such as CVA and ACS were assessed as poor outcomes, the number of events was insufficient to allow statistically robust analyses. Given the clinical importance of cardiovascular risk in AAV, future studies with larger cohorts are warranted to rigorously evaluate the predictive value of baseline indices for cardiovascular events. We also lack a validation cohort. Although the SHAVE cohort is maintained with standardised data collection, future validation in an external or multicentre cohort is needed to confirm the generalisability of our findings. Additionally, the retrospective study method and a single-centre study are inherent limitations of this study. A future prospective study enrolling more patients from more centres will provide more reliable and stable information on the ability of a formula consisting of FFS and eVDI at AAV diagnosis to predict all-cause mortality and progression to ESKD during follow-up in patients with AAV.

## 5 Conclusion

This study developed a formula consisting of FFS and eVDI at AAV diagnosis and demonstrated that it could predict not only all-cause mortality but also ESKD progression during follow-up in patients with AAV.

## Data Availability

The raw data supporting the conclusions of this article will be made available by the authors, without undue reservation.
