# Supplementary figures and images for: A new formula consisting of the five-factor score and earliest vasculitis damage index at diagnosis for predicting poor outcomes of antineutrophil cytoplasmic antibody-associated vasculitis
Source: Front Med (Lausanne). 2025 Aug 6;12:1582892. doi: 10.3389/fmed.2025.1582892 (PMC12364846; doi:10.3389/fmed.2025.1582892)

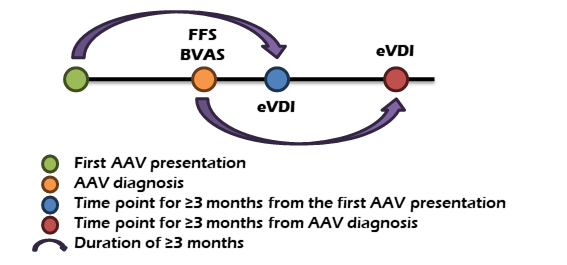

Supplement: Supplementary file 1 [file Image_1.TIF]

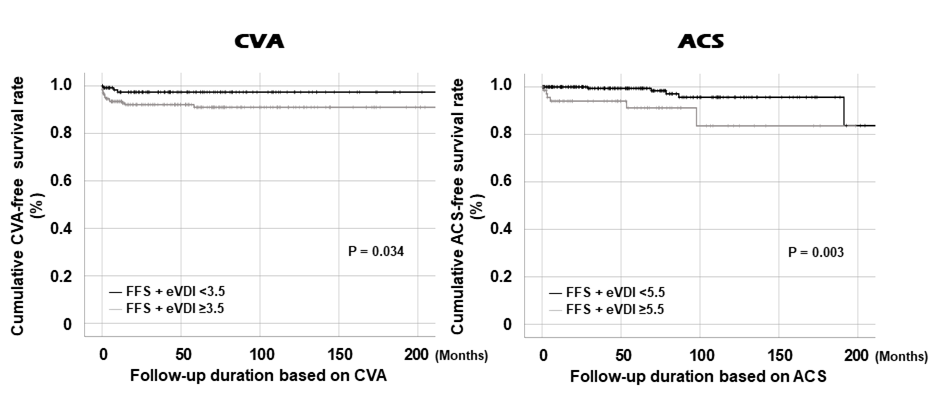

Supplement: Supplementary file 2 [file Image_2.TIF]

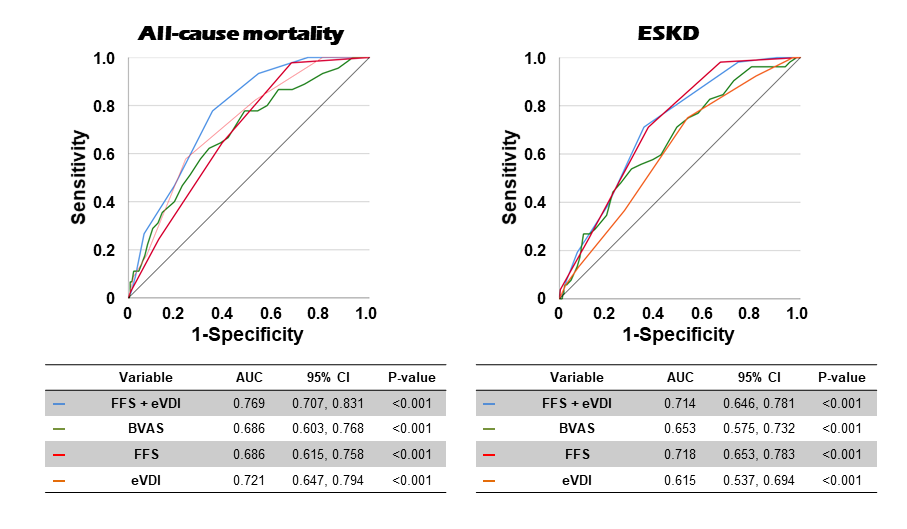

Supplement: Supplementary file 3 [file Image_3.TIF]

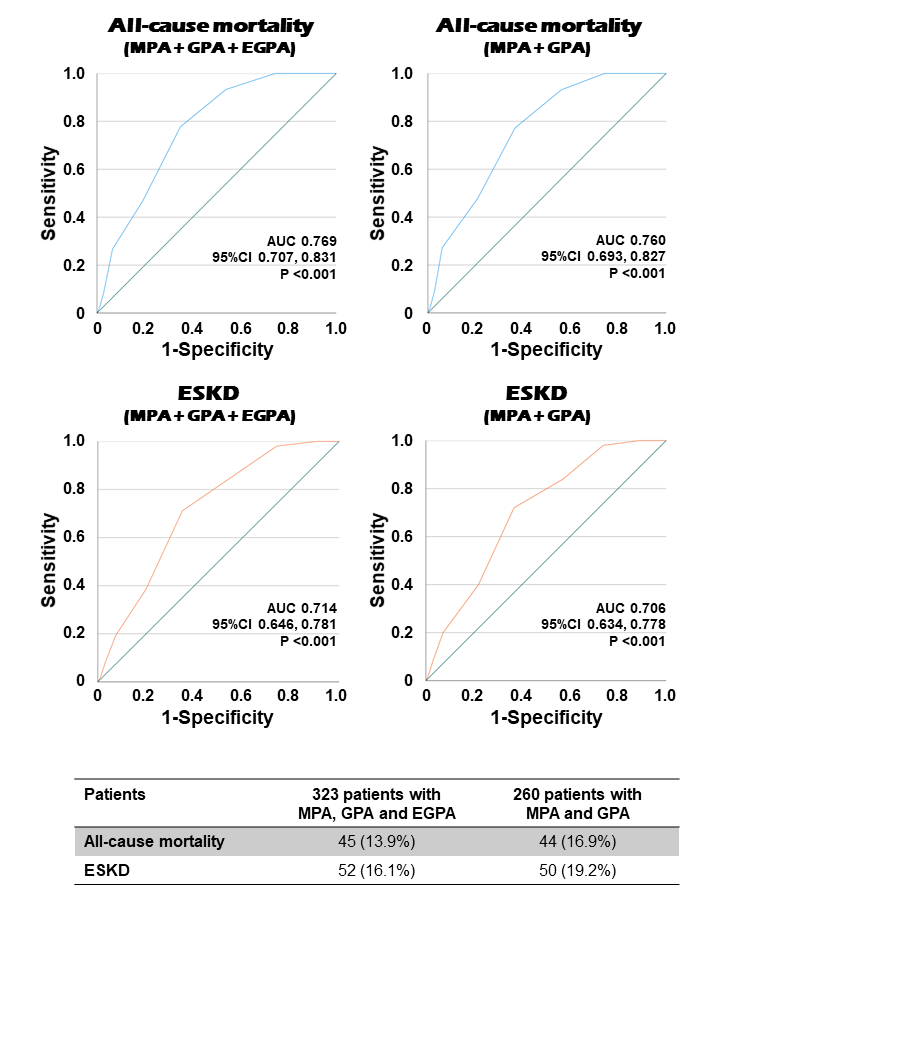

Supplement: Supplementary file 4 [file Image_4.TIF]
