# Supplementary material for: A new formula consisting of the five-factor score and earliest vasculitis damage index at diagnosis for predicting poor outcomes of antineutrophil cytoplasmic antibody-associated vasculitis
Source: Front Med (Lausanne). 2025 Aug 6;12:1582892. doi: 10.3389/fmed.2025.1582892 (PMC12364846; doi:10.3389/fmed.2025.1582892)
Supplement: Supplementary file 5 [file Table_1.DOCX]

**Supplementary Table 1. Univariable Cox proportional hazard analyses of variables at diagnosis for all-cause mortality during follow-up in AAV patients**

| **Variables** | **Univariable** | | |
| --- | --- | --- | --- |
|  | **HR** | **95% CI** | **P value** |
| Age | 1.060 | 1.031, 1.090 | <0.001 |
| Male sex | 1.988 | 1.100, 3.595 | 0.023 |
| BMI | 0.970 | 0.888, 1.058 | 0.490 |
| Ex-smoker | 1.606 | 0.388, 6.650 | 0.514 |
| MPO-ANCA (or P-ANCA) | 1.351 | 0.703, 2.597 | 0.367 |
| PR3-ANCA (or C-ANCA) | 0.742 | 0.313, 1.756 | 0.497 |
| *BVAS* | *1.091* | *1.049, 1.135* | *<0.001* |
| *FFS* | *1.957* | *1.470, 2.606* | *<0.001* |
| *eVDI* | *1.597* | *1.291, 1.976* | *<0.001* |
| ESR | 1.008 | 1.000, 1.016 | 0.037 |
| CRP | 1.008 | 1.004, 1.012 | <0.001 |
| White blood cell count | 1.000 | 1.000, 1.000 | 0.087 |
| Haemoglobin | 0.752 | 0.655, 0.864 | <0.001 |
| Platelet count | 1.000 | 0.998, 1.002 | 0.959 |
| Fasting glucose | 1.005 | 0.999, 1.010 | 0.096 |
| Blood urea nitrogen | 1.013 | 1.005, 1.020 | 0.001 |
| Serum creatinine | 1.147 | 1.029, 1.279 | 0.013 |
| Serum total protein | 0.577 | 0.411, 0.809 | 0.001 |
| Serum albumin | 0.385 | 0.259, 0.573 | <0.001 |
| Hypertension | 1.391 | 0.773, 2.502 | 0.270 |
| T2DM | 1.158 | 0.614, 2.185 | 0.651 |
| Dyslipidaemia | 2.039 | 1.066, 3.902 | 0.031 |
| **FFS + eVDI** | 1.538 | 1.322, 1.790 | **<0.001** |

AAV: ANCA-associated vasculitis; ANCA: antineutrophil cytoplasmic antibody; BMI: body mass index; MPO: myeloperoxidase; P: perinuclear; PR3: proteinase 3; C: cytoplasmic; BVAS: the Birmingham vasculitis activity score; FFS: the five-factor score; eVDI: the earlies vasculitis damage index; ESR: erythrocyte sedimentation rate; CRP: C-reactive protein; T2DM: type 2 diabetes mellitus.
