# Supplementary material for: A new formula consisting of the five-factor score and earliest vasculitis damage index at diagnosis for predicting poor outcomes of antineutrophil cytoplasmic antibody-associated vasculitis
Source: Front Med (Lausanne). 2025 Aug 6;12:1582892. doi: 10.3389/fmed.2025.1582892 (PMC12364846; doi:10.3389/fmed.2025.1582892)
Supplement: Supplementary file 6 [file Table_2.DOCX]

**Supplementary Table 2. Univariable Cox proportional hazard analyses of variables at diagnosis for ESKD during follow-up in AAV patients**

| **Variables** | **Univariable** | | |
| --- | --- | --- | --- |
|  | **HR** | **95% CI** | **P value** |
| Age | 1.025 | 1.003, 1.047 | 0.025 |
| Male sex | 0.888 | 0.491, 1.607 | 0.695 |
| BMI | 0.883 | 0.810, 0.962 | 0.005 |
| Ex-smoker | 0.673 | 0.093, 4.874 | 0.695 |
| MPO-ANCA (or P-ANCA) | 2.747 | 1.291, 5.844 | 0.009 |
| PR3-ANCA (or C-ANCA) | 0.624 | 0.266, 1.461 | 0.277 |
| *BVAS* | *1.072* | *1.034, 1.113* | *<0.001* |
| *FFS* | *2.037* | *1.564, 2.652* | *<0.001* |
| *eVDI* | *1.291* | *1.059, 1.574* | *0.011* |
| ESR | 1.004 | 0.997, 1.011 | 0.248 |
| CRP | 1.003 | 0.999, 1.008 | 0.145 |
| White blood cell count | 1.000 | 1.000, 1.000 | 0.808 |
| Haemoglobin | 0.664 | 0.579, 0.762 | <0.001 |
| Platelet count | 0.998 | 0.995, 1.000 | 0.053 |
| Fasting glucose | 1.002 | 0.997, 1.008 | 0.412 |
| Blood urea nitrogen | 1.031 | 1.025, 1.038 | <0.001 |
| Serum creatinine | 1.533 | 1.425, 1.649 | <0.001 |
| Serum total protein | 1.018 | 0.966, 1.072 | 0.513 |
| Serum albumin | 0.580 | 0.409, 0.822 | 0.002 |
| Hypertension | 2.380 | 1.361, 4.165 | 0.002 |
| T2DM | 0.952 | 0.508, 1.786 | 0.878 |
| Dyslipidaemia | 1.182 | 0.592, 2.359 | 0.635 |
| **FFS + eVDI** | 1.406 | 1.223, 1.617 | <0.001 |

ESKD: end-stage kidney disease; AAV: ANCA-associated vasculitis; ANCA: antineutrophil cytoplasmic antibody; BMI: body mass index; MPO: myeloperoxidase; P: perinuclear; PR3: proteinase 3; C: cytoplasmic; BVAS: the Birmingham vasculitis activity score; FFS: the five-factor score; eVDI: the earlies vasculitis damage index; ESR: erythrocyte sedimentation rate; CRP: C-reactive protein; T2DM: type 2 diabetes mellitus.
