# Supplementary material for: A new formula consisting of the five-factor score and earliest vasculitis damage index at diagnosis for predicting poor outcomes of antineutrophil cytoplasmic antibody-associated vasculitis
Source: Front Med (Lausanne). 2025 Aug 6;12:1582892. doi: 10.3389/fmed.2025.1582892 (PMC12364846; doi:10.3389/fmed.2025.1582892)
Supplement: Supplementary file 7 [file Table_3.DOCX]

**Supplementary Table 3. Comparison of characteristics between surviving and deceased patients**

| **Variables** | **Surviving patients**  **(N=278)** | **Deceased patients**  **(N=45)** | **P-value** |
| --- | --- | --- | --- |
| **Demographic data** |  |  |  |
| Age (years) | 59.0 (19.0) | 66.5 (19.0) | 0.001 |
| Male sex (N, (%)) | 96 (34.5) | 21 (46.7) | 0.116 |
| BMI (kg/m^2^) | 22.5 (4.3) | 22.1 (5.2) | 0.289 |
| Ex-smoker (N, (%)) | 7 (2.5) | 2 (4.4) | 0.364 |
| **ANCA type and positivity (N, (%))** |  |  |  |
| MPO-ANCA (or P-ANCA) positivity | 194 (69.8) | 32 (71.1) | 0.857 |
| PR3-ANCA (or C-ANCA) positivity | 45 (16.2) | 6 (13.3) | 0.626 |
| **AAV-specific indices** |  |  |  |
| BVAS | 11.0 (11.0) | 16.5 (10.0) | <0.001 |
| FFS | 1.0 (2.0) | 2.0 (1.8) | <0.001 |
| eVDI**^*^** | 3.0 (1.0) | 4.0 (1.0) | <0.001 |
| **New equations using AAV-specific indices** |  |  |  |
| BVAS + FFS + eVDI | 14.0 (11.8) | 22.5 (11.8) | <0.001 |
| BVAS + FFS | 12.0 (12.0) | 19.0 (10.8) | <0.001 |
| BVAS + eVDI | 13.0 (11.8) | 20.5 (10.8) | <0.001 |
| FFS + eVDI | 4.0 (3.0) | 5.0 (2.0) | <0.001 |
| **Acute phase reactants** |  |  |  |
| ESR (mm/hr) | 54.0 (73.8) | 70.5 (86.5) | 0.069 |
| CRP (mg/L) | 7.2 (47.6) | 50.8 (103.4) | <0.001 |
| **Laboratory results** |  |  |  |
| White blood cell count (/mm^3^) | 8,795.0 (5,990.0) | 11,455.0 (7,692.0) | 0.028 |
| Haemoglobin (g/dL) | 12.0 (3.4) | 10.0 (3.0) | <0.001 |
| Platelet count (× 1000/mm^3^) | 293.0 (153.3) | 315.0 (189.8) | 0.564 |
| Fasting glucose (mg/dL) | 101.5 (27.8) | 99.5 (46.3) | 0.255 |
| Blood urea nitrogen (mg/dL) | 17.0 (14.2) | 31.0 (39.6) | <0.001 |
| Serum creatinine (mg/dL) | 0.8 (0.7) | 1.8 (2.4) | <0.001 |
| Serum total protein (g/dL) | 6.8 (1.3) | 6.4 (1.3) | <0.001 |
| Serum albumin (g/dL) | 3.8 (1.1) | 3.2 (1.3) | <0.001 |
| **Comorbidities (N, (%))** |  |  |  |
| T2DM | 67 (24.1) | 14 (31.1) | 0.314 |
| Hypertension | 109 (39.2) | 23 (51.1) | 0.132 |
| Dyslipidaemia | 40 (14.4) | 13 (28.9) | 0.015 |

Values are expressed as a median (interquartile range) or N (%).

eVDI*: the earliest VDI was defined as follows: the first VDI assessed at more than 3 months after AAV diagnosis or at more than 3 months after the first presentation of AAV-related manifestations.

AAV: ANCA-associated vasculitis; ANCA: antineutrophil cytoplasmic antibody; BMI: body mass index; MPA: microscopic polyangiitis; GPA: granulomatosis with polyangiitis; EGPA: eosinophilic granulomatosis with polyangiitis; MPO: myeloperoxidase; P: perinuclear; PR3: proteinase 3; C: cytoplasmic; BVAS: the Birmingham vasculitis activity score; FFS: the five-factor score; eVDI: the earlies vasculitis damage index; ESR: erythrocyte sedimentation rate; CRP: C-reactive protein; T2DM: type 2 diabetes mellitus.
