# Supplementary material for: A new formula consisting of the five-factor score and earliest vasculitis damage index at diagnosis for predicting poor outcomes of antineutrophil cytoplasmic antibody-associated vasculitis
Source: Front Med (Lausanne). 2025 Aug 6;12:1582892. doi: 10.3389/fmed.2025.1582892 (PMC12364846; doi:10.3389/fmed.2025.1582892)
Supplement: Supplementary file 8 [file Table_4.DOCX]

**Supplementary Table 4. Comparison of characteristics between patients with ESKD and those without**

| **Variables** | **Patients without**  **ESKD (N=271)** | **Patients with**  **ESKD (N=52)** | **P-value** |
| --- | --- | --- | --- |
| **Demographic data** |  |  |  |
| Age (years) | 60.0 (19.0) | 62.0 (19.0) | 0.082 |
| Male sex (N, (%)) | 101 (37.3) | 16 (30.8) | 0.372 |
| BMI (kg/m^2^) | 22.7 (4.6) | 21.1 (3.7) | <0.001 |
| Ex-smoker (N, (%)) | 8 (3.0) | 1 (1.9) | 1.000 |
| **ANCA type and positivity (N, (%))** |  |  |  |
| MPO-ANCA (or P-ANCA) positivity | 182 (67.2) | 44 (84.6) | 0.012 |
| PR3-ANCA (or C-ANCA) positivity | 45 (16.6) | 6 (11.5) | 0.359 |
| **AAV-specific indices** |  |  |  |
| BVAS | 11.0 (11.0) | 16.0 (12.5) | <0.001 |
| FFS | 1.0 (2.0) | 2.0 (2.0) | <0.001 |
| eVDI**^*^** | 3.0 (2.0) | 3.0 (2.0) | 0.007 |
| **New equations using AAV-specific indices** |  |  |  |
| BVAS + FFS + eVDI | 14.0 (12.0) | 22.0 (13.5) | <0.001 |
| BVAS + FFS | 12.0 (12.0) | 19.0 (13.0) | <0.001 |
| BVAS + eVDI | 14.0 (12.0) | 20.0 (12.5) | <0.001 |
| FFS + eVDI | 4.0 (3.0) | 5.0 (2.0) | <0.001 |
| **Acute phase reactants** |  |  |  |
| ESR (mm/hr) | 54.0 (77.0) | 68.0 (58.5) | 0.283 |
| CRP (mg/L) | 9.1 (58.1) | 20.2 (75.4) | 0.012 |
| **Laboratory results** |  |  |  |
| White blood cell count (/mm^3^) | 9,180.0 (6,050.0) | 9,280.0 (7,410.0) | 0.612 |
| Haemoglobin (g/dL) | 12.0 (3.2) | 9.3 (2.8) | <0.001 |
| Platelet count (× 1000/mm^3^) | 308.0 (162.0) | 255.0 (140.0) | 0.006 |
| Fasting glucose (mg/dL) | 102.0 (27.0) | 97.0 (38.5) | 0.672 |
| Blood urea nitrogen (mg/dL) | 16.2 (10.9) | 41.7 (25.2) | <0.001 |
| Serum creatinine (mg/dL) | 0.8 (0.6) | 4.4 (3.7) | <0.001 |
| Serum total protein (g/dL) | 6.8 (1.3) | 6.3 (1.2) | <0.001 |
| Serum albumin (g/dL) | 3.8 (1.1) | 3.3 (0.9) | <0.001 |
| **Comorbidities (N, (%))** |  |  |  |
| T2DM | 68 (25.1) | 13 (25.0) | 0.989 |
| Hypertension | 100 (36.9) | 32 (61.5) | <0.001 |
| Dyslipidaemia | 43 (15.9) | 10 (19.2) | 0.549 |

Values are expressed as a median (interquartile range) or N (%).

eVDI*: the earliest VDI was defined as follows: the first VDI assessed at more than 3 months after AAV diagnosis or at more than 3 months after the first presentation of AAV-related manifestations.

AAV: ANCA-associated vasculitis; ANCA: antineutrophil cytoplasmic antibody; BMI: body mass index; MPA: microscopic polyangiitis; GPA: granulomatosis with polyangiitis; EGPA: eosinophilic granulomatosis with polyangiitis; MPO: myeloperoxidase; P: perinuclear; PR3: proteinase 3; C: cytoplasmic; BVAS: the Birmingham vasculitis activity score; FFS: the five-factor score; eVDI: the earlies vasculitis damage index; ESR: erythrocyte sedimentation rate; CRP: C-reactive protein; T2DM: type 2 diabetes mellitus.
